# PIWI-like protein, HIWI2 is aberrantly expressed in retinoblastoma cells and affects cell-cycle potentially through OTX2

**DOI:** 10.1186/s11658-017-0048-y

**Published:** 2017-08-29

**Authors:** Suganya Sivagurunathan, Jayamuruga Pandian Arunachalam, Subbulakshmi Chidambaram

**Affiliations:** 1 RS Mehta Jain Department of Biochemistry and Cell Biology, Vision Research Foundation, Chennai, India; 2 School of Chemical and Biotechnology, SASTRA University, Thanjavur, India; 3 SN ONGC Department of Genetics and Molecular Biology, Vision Research Foundation, Chennai, India; 4 Central Inter-Disciplinary Research Facility (CIDRF), Sri Balaji Vidyapeeth University, Mahatma Gandhi Medical College and Research Institute Campus, Pondicherry, India; 5 Department of Biochemistry and Molecular Biology, Pondicherry University, Puducherry, India

**Keywords:** Y79, ARPE19, PCNA, Stem cell markers

## Abstract

Retinoblastoma (RB), a childhood cancer, is caused by biallelic mutation of the *RB1* gene, but its development is not clearly understood. Furthermore, the presence of a cancer stem cell subpopulation in RB might impact its treatment. PIWI protein, known for its role in stem cell self-renewal, is aberrantly expressed in cancers. We examined the role of the PIWI-like protein HIWI2 in RB and its effect on the stem cell markers in cells of the RB line, Y79. The expression of HIWI2 is significantly increased in Y79 compared with its level in HeLa and ARPE19 cells. The stem cell markers Oct-3/4, Nanog and Sox-2 were not altered upon HIWI2 knockdown in Y79 cells. Interestingly, OTX2 was significantly downregulated in the absence of HIWI2. *Otx2* transcripts also decreased in HIWI2-silenced Y79 and ARPE19 cells. Moreover, silencing HIWI2 in Y79 accumulated the cells at G2–M phase and reduced the levels of proliferating cell nuclear antigen (PCNA) and the tumor suppressor, p16. Our results demonstrate that HIWI2 is aberrantly expressed in Y79 cells and silencing of HIWI2 downregulates OTX2, suggesting that HIWI2 might play a role in the progression of RB.

## Introduction

Retinoblastoma (RB), the most common ocular cancer, mainly occurs due to the biallelic mutation of the *RB1* gene on chromosome 13 [1]. The “two-hit” hypothesis suggests that two mutational events are needed in *RB1* for RB to develop [2]. Besides the inactivation of *RB1*, epigenetic changes are also involved in the tumor formation [3, 4].

Although significant advances have been made in recent years, the mechanism behind the onset and progression of RB is still not well understood. Because many factors are involved in its development, RB treatment is challenging. Current therapies like enucleation, brachytheraphy and laser photocoagulation have devastating effects [5], so better therapies with less disease morbidity are essential. Designing such therapies requires the identification of endogenous proteins that can be modulated to curb the progression of RB.

PIWI-like proteins are essential for stem cell self-renewal [6]. They bind with small non-coding RNAs called piwi-interacting RNAs (piRNAs) [7]. These PIWI–piRNA complexes mainly function in silencing transposable elements to protect the genomic integrity of germline cells [8]. HIWI, HILI, PIWIL3 and HIWI2 are the four members of the PIWI family in humans [9]. Besides their role in stem cell self-renewal and transposon silencing in germline cells, they are important in epigenetic silencing [10].

We recently demonstrated the function of HIWI2 in somatic cells, where it plays a role in maintaining the integrity of retinal pigment epithelial cells by regulating the Akt/GSK3 pathway [11]. Other recent reports have shown the abnormal expression of PIWI-like proteins in various cancers, where they are known to promote cell division, resist apoptosis and facilitate cell invasion and migration [12]. PIWI-like proteins are known to influence cancer cells through modulation of STAT3, TGFβ, FGF, MAPK-ERK and p53 signalling pathways [13, 14]. PIWI-like proteins and piRNAs are also suggested as novel prognostic biomarkers in breast cancer [15] and gastric cancer [16]. Although there has been considerable study of the role of PIWI-like proteins in cancer, their involvement in RB remains unexplored.

Recent studies suggest cancer stem cells as one of the reason for the resistance and recurrence of the tumor [17], so the genes responsible for stemness are candidates for therapy [18]. Aberrant expression of PIWI-like proteins in cancerous conditions indicate their role in the stem cell characteristics of RB.

In this study, we examined the role of HIWI2 in RB and its effect on the stem cell characteristics of RB cells. Our results show that HIWI2 is highly expressed in Y79 cells and affects cell cycle, possibly through OTX2.

## Experimental procedures

### Cell culture

Y79 cells were cultured using RPMI 1640 (GE Healthcare Life Sciences) supplemented with 10% (*v*/v) heat-inactivated fetal bovine serum (FBS; Invitrogen). The cells were maintained in 5% CO_2_ at 37 °C with antibiotics and antimycotics (Thermo Fisher Scientific). ARPE19 cells were maintained in DMEM-F12 (Sigma-Aldrich) and 14.2 mM sodium bicarbonate and HeLa cells in DMEM-HG (HIMEDIA) with 10% (*v*/v) heat-inactivated FBS under the same conditions.

### RNAi experiments

The cells were grown to confluency and 2 × 10^5^ cells were seeded in 6-well plates. DsiRNA specific for HIWI2 (sense strand 5′-GCAUCACUAGAUGGACAAUCCAAGA-3′; antisense3’-ACCGUAGUGAUCUACCUGUUAGGUUCU-5′; Integrated DNA Technologies) was transfected to the cells using Lipofectamine RNAiMax (Invitrogen) as per the manufacturer’s protocol. Protein lysates were obtained by lysing the cells 48 h post transfection.

### Real-time PCR

RNA was isolated from cells transfected with scrambled siRNA (Si-Control) and HIWI2 siRNA (Si-HIWI2) 48 h post transfection using TRI reagent (Sigma-Aldrich). 1 μg of RNA was converted to cDNA using an iScript kit (Biorad). The transcript levels of *Otx2* and *HIWI2* were determined using SYBR green assays (Roche Diagnostics) via real-time PCR (Roche Diagnostics). *18S rRNA* and *β-actin* were used as the endogenous controls. The gene expression of the transcripts was quantified using the relative quantification method.

### Western blot

Cells were lysed using radio immunoprecipitation assay (RIPA) buffer consisting of 150 mM NaCl, 0.1%TritonX-100, 0.5% sodium deoxycholate, 0.1% SDS and 50 mM Tris (pH 8.0) with protease inhibitors (1 mmol/l dithiothreitol, 0.5 mmol/l phenylmethylsulfonyl fluoride, 1 mg/ml leupeptin, 10 mmol/l p-nitrophenylphosphate, 10 mmol/l h-glycerol phosphate). Then, the cells were sonicated. The lysate was centrifuged at 10,000 rpm for 10 min, the protein concentration was estimated using BCA protein assay reagent (Thermo Scientific), and 50 μg of protein was resolved on SDS-PAGE gel and electrotransferred to nitrocellulose membrane (GE Healthcare). The blots were incubated in blocking buffer (5% skimmed milk powder in Tris-buffered saline) for 1 h and were probed against HIWI2 (Pierce), OTX2 (Abcam), p16 (PathnSitu Biotechnologies) and PCNA (Cell Signaling Technology) primary antibodies in a 1:1000 dilution of blocking buffer. Anti-rabbit and anti-mouse secondary antibodies (Santa Cruz Biotechnology) were used in a 1:10,000 dilution. The blots were then developed with FluorChem FC3 (Protein Simple) using ECL reagent (GE Healthcare).

### Proteome profiler array

Proteins that were altered after HIWI2 silencing were screened using the Human Pluripotent Stem Cell Array Kit (R&D Systems). Protein lysates were prepared according to manufacturer’s protocol and 200 μg of protein was used for the array. The array was imaged and quantified using the Alpha View software (ProteinSimple). Fold changes in the protein expression levels are represented.

### Cell cycle analysis

HIWI2-silenced Y79 cells were washed twice with phosphate buffered saline (PBS) by centrifuging at 1500 rpm for 5 min. The washed cells were fixed with 30% ice-cold ethanol via incubation for 30 min on ice. After fixation, the cells were again washed with PBS by centrifuging at 3000 rpm for 5 min. Cells were treated with 0.5 mg/ml RNase A (Sigma Aldrich) by incubating at 37 °C for 20 min. They were then stained with 50 μg/ml propidium iodide (Sigma Aldrich) for 30 min at 4 °C. The stained cells were analysed using FACSCalibur (Beckton Dickinson). A total of 20,000 events were collected for each sample.

### Statistical analysis

Student’s *t*-test was used to comparatively analyse the mean obtained from three independent experiments. The difference between the samples were considered to be statistically significant when the *p*-values were *p* < 0.05.

## Results

### The expression of HIWI2 is elevated in Y79 cells

**Fig. 1:**
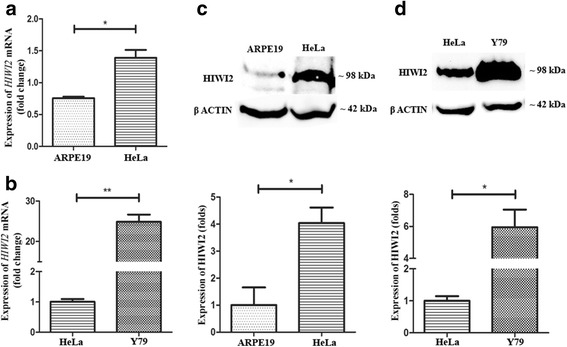
HIWI2 is aberrantly expressed in retinoblastoma. **a**, **b** – Real-time PCR shows the expression of *HIWI2* transcripts in ARPE19, HeLa and Y79 cell lines. *β-actin* was used for normalization and fold changes in expression are indicated. **c** – Western blot shows the expression of HIWI2 in protein lysates of ARPE19 and HeLa. β-ACTIN was used for normalization and the fold changes are indicated. The bar graph represents the quantification of the western blot image representing the fold change in the expression of HIWI2 in ARPE19 and HeLa cells. **d** – Western blot shows the expression of HIWI2 in protein lysates of HeLa and Y79 cell lines. β-ACTIN was used for normalization and the fold changes are indicated. The bar graph represents the quantification of the western blot image representing the fold change of HIWI2 in HeLa and Y79 cell lines. Student’s t-test was used for statistical analysis. *p < 0.05 and **p < 0.01 were considered statistically significant

The expression of the *HIWI2* transcript was studied using quantitative PCR in human retinal pigment epithelial cells (ARPE19), human cervical epithelial carcinoma cells (HeLa) and human RB cells (Y79). The expression of *HIWI2* was 1.38-fold higher in HeLa than in ARPE19 (Fig. 1a). Interestingly, Y79 showed a 24.86-fold increase when HeLa is considered (Fig. 1b).

Western blot analysis showed that HIWI2 expression was 4-fold higher in HeLa than in ARPE19 (Fig. 1c) and there was 5.9-fold increase in HIWI2 expression in Y79 over HeLa (Fig. 1d). Clearly, HIWI2 is aberrantly expressed in Y79 cells at both the mRNA and protein levels.

### OTX2 is downregulated upon HIWI2 knockdown

**Fig. 2:**
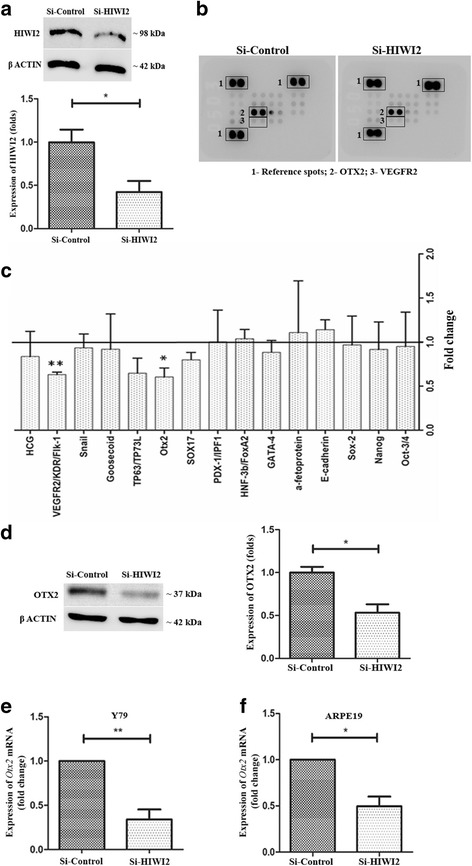
OTX2 is downregulated upon HIWI2 knockdown. **a** – Representative western blot image showing HIWI2 expression level in Si-HIWI2 Y79 cells. β-ACTIN was used for normalization and the fold changes are indicated. **b** – Representative proteome profiler array blot used for screening Si-Control and Si-HIWI2 Y79 protein lysates. **c** – Fold changes in the expressions of the proteins that are altered in Si-HIWI2 Y79 cells obtained from quantification of the array, are represented in the graph. **d** – Representative western blot image showing OTX2 expression level in Si-HIWI2 Y79 cells. β-ACTIN was used for normalization and the fold changes are indicated. **e** – Real-time PCR results indicating the fold changes in the levels of *Otx2* transcript in Si-HIWI2 Y79 cells. **f** – Real-time PCR results showing the reduced expression of *Otx2* transcripts in Si-HIWI2 ARPE19 cells. Student’s *t*-test was used for statistical analysis. *p < 0.05 and **p < 0.01 were considered statistically significant

To discern the role of HIWI2 on stem cell markers of RB, HIWI2 was silenced in Y79 cells using DsiRNA (Fig. 2a). The cell lysates were screened using a Human Pluripotent Stem Cell Array (Fig. 2b). Interestingly, OTX2, an eye field specification transcription factor, and VEGFR2 were significantly downregulated in Si-HIWI2 cells (Fig. 2b and c). However, Oct-3/4, Nanog and Sox-2 did not show any significant changes in HIWI2-silenced Y79 cells (Fig. 2b and c).

HIWI2 has been reported to affect the invasive properties of HeLa [19], which might be attributed to VEGF and its receptors. Since OTX2 plays crucial roles in the development of the eye [20], we further evaluated the relationship between OTX2 and HIWI2. OTX2 expression in Si-HIWI2 cells decreased 1.87-fold, which further validated the stem cell array data (Fig. 2d). The mRNA expression of *Otx2* in HIWI2-silenced cells was also in accordance with the results obtained in the array (Fig. 2e). The expression of *Otx2* in Si-HIWI2 cells was 2.94-fold lower than in Si-Control cells (Fig. 2e). Since knockout of *otx2* has shown to affect retinal pigment epithelial function [21], the expression of *Otx2* transcripts were also evaluated in ARPE19. On silencing HIWI2, *Otx2* was found to be 2.02-fold reduced (Fig. 2f). Thus, the absence of HIWI2 in Y79 cells specifically downregulated OTX2 and showed no significant effect on other stem cell genes tested.

### Suppression of HIWI2 decreases proliferation of Y79 cells

**Fig. 3:**
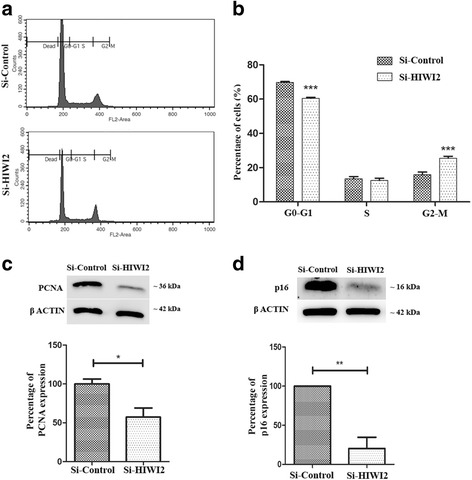
HIWI2 silencing affects the proliferation of Y79 cells. **a** – Histogram shows the changes in the cell cycle distribution of Si-HIWI2 Y79 cells. **b** – Bar graph represents the percentage of cells distributed among various phases of the cell cycle in HIWI2 knockdown. **c** – Western blot showing the expression of PCNA upon HIWI2 knockdown in Y79 cells. Bar graph represents the percentage of PCNA expression. **d** – Western blot showing the expression of p16 upon HIWI2 knockdown in Y79 cells. The bar graph represents the percentage of p16 expression. Student’s *t*-test was used for statistical analysis. *p < 0.05, **p < 0.01 and ***p < 0.001 were considered as statistically significant

Since OTX2 directly regulates cell cycle genes [22], we monitored the effects of HIWI2 on the cell cycle distribution of Y79 cells. Cell cycle analysis by flow cytometry indicated that suppression of HIWI2 affects the cell cycle in Y79 cells (Fig. 3a). HIWI2-silenced Y79 cells were arrested at G2–M phase (Fig. 3b). The expression of PCNA significantly decreased by 43% in Si-HIWI2 cells, reflecting the effect of HIWI2 on the proliferation of Y79 cells (Fig. 3c).

Since HIWI2 is known to regulate the expression of p16 [10], a cell cycle regulator, we evaluated p16 levels in Si-HIWI2 cells. Silencing HIWI2 reduces the expression of p16 (Fig. 3d). These results indicate that HIWI2 alters the cell cycle distribution of Y79 cells, and therefore could affect proliferation.

## Discussion

This study reports the first evidence of elevated expression of HIWI2 in Y79 cells and a possible functional relationship with OTX2. Furthermore, a projected role of HIWI2 in cell cycle regulation and proliferation of Y79 cells has been investigated. Our results show that HIWI2 might play a significant role in the progression of RB.

The expression of PIWI-like proteins was earlier reported to be upregulated in various cancer conditions [16, 23] and was observed to increase with increasing tumor grades [24]. Wang et al. showed that the suppression of HIWI proteins reduces growth, invasion and migration in glioma cells [25]. Our study also shows an elevated expression of HIWI2 in Y79 cells. Though increased expression of HIWI2 is reported in cervical cancer tissues, Y79 showed higher expression of HIWI2 than the levels reported in cervical cancer cells [19]. This could be attributed to the cellular origin of cancer, which correlates with the finding from our previous study showing higher expression of PIWI-like proteins in the retina than in the retinal pigment epithelium (RPE) [11].

piRNAs, the small noncoding RNAs that bind to PIWI proteins, are also deregulated in cancerous conditions. The expression of piR651 significantly increases in non-small cell lung carcinoma [26] and piRNA823 is overexpressed in multiple myeloma patients [27]. Hence, it is possible that HIWI2-associated piRNAs might also be altered in RB and could play a role in the pathogenesis.

Recently, cancer stem cells (CSC) and markers have become a promising therapeutic target in various cancer types. For instance, the polycomb family protein BMI-1, designated as a stemness marker for CSC, is important for the self-renewal and tumor initiation of CSCs, and also for neural and hematopoietic stem cells. Interestingly, BMI-1 also targets the p16^INK4a^ gene to regulate various events of tumor progression [28]. Similarly, PIWI proteins, which are important for germline stem cell (GSC) self-renewal [6], are also needed for continued proliferation of cancer cells [18]. Hence, we could speculate that PIWI-like proteins might be required for CSC self-renewal as well, which remains unexplored.

The stem cell-related genes in cancer cells could define the characteristics of CSCs. In Y79, the absence of HIWI2 deregulated OTX2, an eye field specification marker that is critical for the development of forebrain and eye [20]. OTX2 is crucial for cell fate determination and terminal differentiation of photoreceptors [29]. Deletion of *otx2* in adult mouse retina causes rapid changes in RPE, and degeneration of the outer segments of photoreceptors [21]. *otx2* gene mutations are associated with severe ocular malformations. This includes anopthalmia, micropthalmia, coloboma and other retinal dystrophy [30, 31]. Taken together, HIWI2 regulation on OTX2 indicates an important role for HIWI2 in human retinal physiology.

HIWI2 might regulate OTX2 either directly or indirectly through signalling pathways. In our previous report, we demonstrated the effects of HIWI2 silencing on the Akt pathway [11]. OTX2 is known to be activated by the PI3K/Akt signalling axis [32]. In addition, FGF2 increases the expression of OTX2 in precursor cells of mammalian telencephalon [33] and OTX2 is also known to regulate FGF8 and other FGF signalling responses in mice [34]. Interestingly, HIWI2 is also known to activate the FGF signalling pathway in breast cancer cells [14]. Perhaps, HIWI2 might regulate the expression of OTX2 either through PI3K/Akt or FGF signalling pathways.

VEGF and its receptor VEGFR2 are known to increase with invasiveness of RB [35]. Intriguingly, downregulating the expression of piRNA-823 reduces VEGF levels in multiple myeloma [27]. Our results show that the VEGFR2 is downregulated in RB cells on HIWI2 silencing. Moreover, bevacizumab, an anti-VEGF agent is known to inhibit the differentiation of RB cells [36]. OTX2 is also involved in inhibiting the differentiation of medulloblastoma cells [22]. Since the levels of both OTX2 and VEGFR2 are altered upon HIWI2 knockdown, it would be interesting to study whether HIWI2 affects the differentiation of RB cells.

We also observed that HIWI2 silencing arrests RB cells at the G2–M phase. However, it has been reported that loss of HIWI2 in HeLa did not show any difference in cell cycle distribution [19]. Therefore, the effects of HIWI2 on cell cycle distribution might be cell specific, as HIWI knockdown arrested gastric cancer cells at the G2–M stage, whereas glioma cells were arrested at the G0–G1 stage [25, 37].

In addition, a low level of PCNA in Y79 cells upon HIWI2 silencing suggested inhibition of proliferation. Besides, reduced expression of p16 in HIWI2 knockdown cells also indicated suppression of proliferation as it has been reported that downregulation of p16, hinders proliferation in cervical cancer cells [38]. Moreover, in RB patients, the p16 gene is found to be downregulated due to hypermethylation at the p16^INK4a^ promoter region [3]. Since the PIWI/piRNA complex is an epigenetic activator, HIWI2 might have caused a reduction in the expression of p16 in Si-HIWI2 Y79 cells [39]. Nonetheless, determining whether HIWI2 has direct or secondary effects will require further study.

A recent report suggested OTX2 as a probable therapeutic target, since it decreases the tumor size and growth in RB xenografts [40]. In this study, we have shown the effect of HIWI2 in downregulating the expression of OTX2 in Y79. Other reports have shown the importance of HIWI2 in tumorigenesis and it has also been suggested as a biomarker in various cancer conditions [12]. Unravelling the exact molecular mechanism of HIWI2 in RB can determine its potential as a therapeutic target in the future.

## Conclusion

We show that the PIWI-like protein HIWI2 is aberrantly expressed in retinoblastoma cells. Silencing HIWI2 arrests cell cycle probably through OTX2. This study provides novel insights into the possible crosstalk between HIWI2 and OTX2. Our results also point out that HIWI2 might play a crucial role in retinal pathophysiology. Further research could throw light on the potential of HIWI2 as a therapeutic target for retinoblastoma.

## Abbreviations

ARPE19: Adult retinal pigment epithelial cells
CSC: Cancer stem cell
PCNA: Proliferating cell nuclear antigen
RB: Retinoblastoma
RPE: Retinal pigment epithelium
